# Reduced Volume of the Arcuate Fasciculus in Adults with High-Functioning Autism Spectrum Conditions

**DOI:** 10.3389/fnhum.2016.00214

**Published:** 2016-05-12

**Authors:** Rachel L. Moseley, Marta M. Correia, Simon Baron-Cohen, Yury Shtyrov, Friedemann Pulvermüller, Bettina Mohr

**Affiliations:** ^1^Department of Psychology, Bournemouth UniversityDorset, UK; ^2^Medical Research Council Cognition and Brain Sciences UnitCambridge, UK; ^3^Brain Mapping Unit, Department of Psychiatry, University of CambridgeCambridge, UK; ^4^Autism Research Centre, Department of Psychiatry, University of CambridgeCambridge, UK; ^5^Cambridge Lifespan Asperger Syndrome Service Clinic, Cambridgeshire and Peterborough National Health Service Foundation TrustCambridge, UK; ^6^Center of Functionally Integrative Neuroscience (CFIN), Department of Clinical Medicine, Aarhus UniversityAarhus, Denmark; ^7^Centre for Cognition and Decision Making, National Research University Higher School of EconomicsMoscow, Russia; ^8^Brain Language Laboratory, Freie Universität BerlinBerlin, Germany; ^9^Department of Psychiatry, Charité–Universitätsmedizin BerlinBerlin, Germany

**Keywords:** autism, Asperger syndrome, diffusion-weighted imaging (DWI), arcuate fasciculus, language

## Abstract

Atypical language is a fundamental feature of autism spectrum conditions (ASC), but few studies have examined the structural integrity of the arcuate fasciculus, the major white matter tract connecting frontal and temporal language regions, which is usually implicated as the main transfer route used in processing linguistic information by the brain. Abnormalities in the arcuate have been reported in young children with ASC, mostly in low-functioning or non-verbal individuals, but little is known regarding the structural properties of the arcuate in adults with ASC or, in particular, in individuals with ASC who have intact language, such as those with high-functioning autism or Asperger syndrome. We used probabilistic tractography of diffusion-weighted imaging to isolate and scrutinize the arcuate in a mixed-gender sample of 18 high-functioning adults with ASC (17 Asperger syndrome) and 14 age- and IQ-matched typically developing controls. Arcuate volume was significantly reduced bilaterally with clearest differences in the right hemisphere. This finding remained significant in an analysis of all male participants alone. Volumetric reduction in the arcuate was significantly correlated with the severity of autistic symptoms as measured by the Autism-Spectrum Quotient. These data reveal that structural differences are present even in high-functioning adults with ASC, who presented with no clinically manifest language deficits and had no reported developmental language delay. Arcuate structural integrity may be useful as an index of ASC severity and thus as a predictor and biomarker for ASC. Implications for future research are discussed.

## Introduction

Communication impairments are archetypal of ASC, with delayed or absent language development the primary cause of concern and referral in many cases (Siegel et al., 1988; De Giacomo and Fombonne, 1998). A significant proportion of individuals with ASC will remain minimally verbal into adulthood (Howlin et al., 2014; Pickles et al., 2014), sometimes presenting with limited to non-speech sounds, stereotyped use of a few words or phrases, and echolalia. Even high-functioning individuals with ASC exhibit a broad range of abnormalities across several major linguistic domains, including prosody, syntax, semantics, and pragmatics (Eigsti et al., 2011; Moseley et al., 2013, 2014, 2015). Although the diagnosis of Asperger syndrome (DSM IV-TR: American Psychiatric Association, 2000), one of the major variants of ASC, was previously given on the basis of typical, non-delayed language development, these individuals may also show receptive and expressive language skills at “well below chronological age level” (Howlin, 2003). They are particularly noted for their use of idiosyncratic, pedantic language, which Hans Asperger described in his “little professor” patients (Asperger, 1944). This particular feature may be the linguistic expression of difficulties with ‘theory of mind’ (inaccurately assessing the knowledge of their listeners), and ‘weak central coherence’ (providing irrelevant and uninformative detail rather than summarizing the ‘gist’ of the matter). These two cognitive accounts are not easily disentangled in the domain of communication, as including too much detail and failing to summarize the wider picture may arise because of a failure to monitor and recognize the listener’s informational needs (Baron-Cohen, 1988). Nevertheless, the neuronal basis of language difficulties in ASC, which seem to affect all linguistic levels (phonological, lexical, syntactic, semantic, and pragmatic), requires further study.

A major white matter tract traditionally implicated in language impairments is the arcuate fasciculus (Geschwind, 1965; Catani and Ffytche, 2005; Ardila, 2010). The properties of this frontotemporal fiber bundle distinguish language-using humans from other non-linguistic primate species (Catani and Ffytche, 2005; Glasser and Rilling, 2008; Rilling et al., 2008; Saur et al., 2008). It consists of a longer, direct segment connecting Wernicke’s area to Broca’s area, and two indirect segments: an anterior part linking Broca’s area with the inferior parietal lobule and a posterior part linking inferior parietal lobule with the superior-temporal gyrus and sulcus (Wernicke’s area; Catani and Mesulam, 2008; Bernal and Ardila, 2009; Bernal and Altman, 2010).

Like language function itself, the arcuate is believed to be left-lateralized in the majority of adults (Catani et al., 2007) and children (Lebel and Beaulieu, 2009). The relationship between *structural* lateralisation of the arcuate and *functional* lateralisation of language is not always transparent (Vernooij et al., 2007; Propper et al., 2010), but its structural properties correlate with behavioral measures of language function, such as word learning (López-Barroso et al., 2013), verbal recall (Catani et al., 2007) and the development of phonological awareness and reading (Yeatman et al., 2011). Although most brain language models assume that the arcuate plays a role in translating acoustic into articulatory linguistic representations (Wernicke, 1874; Geschwind, 1965; Hickok and Poeppel, 2004, 2007), current action-perception theories of language additionally purport that the arcuate is crucial for building linguistic representations at all levels [phonological, lexical, syntactic, semantic and pragmatic (Pulvermüller and Fadiga, 2010)]. This position suggests AF degradation as a likely cause of multi-level language and communication deficits such as those manifest in ASC.

Despite the linguistic relevance of this tract and the prominence of language impairments in ASC diagnosis, few studies have examined the arcuate fasciculus structurally in autism. White matter integrity can be studied non-invasively *in vivo* using diffusion weighted imaging (DWI), which illuminates the microstructure of white-matter tracts by detecting the diffusion of water through brain tissue (Alexander et al., 2007).

Only four previous DWI studies investigated arcuate structure in autistic children in mixed gender groups. Two reported a lack of typical left-hemispheric asymmetry as compared to typically developing controls (Wan et al., 2012; Joseph et al., 2014). Another two reported reduced FA in the left arcuate fasciculus when children with ASC are compared to typically developing controls (Kumar et al., 2010; Lai G. et al., 2012). As Kumar et al. (2010) also included a comparison group of non-autistic children with intellectual disability, they showed that longer fiber length of the right arcuate fasciculus set the ASC group apart from both comparison groups. Ingalhalikar et al. (2011) studied an ASC group consisting of children with mixed language abilities, including language-impaired participants and those in the normal range. They reported reduced FA not in the arcuate but, instead, in the adjacent parts of the superior longitudinal fasciculus (SLF), a linguistically important connection between inferior-frontal and temporo-parietal cortical areas. This finding must be interpreted with caution as it pertains to a more inclusive pathway of which the arcuate is a single part: the SLF contains connections between the frontal, parietal, occipital, and temporal lobes (Schmahmann and Pandya, 2006), the arcuate being sometimes defined as the ‘long segment’ connecting Broca’s and Wernicke’s areas (Liégeois et al., 2013). A fuller description of these studies can be seen in Supplementary Materials.

It is difficult to interpret the findings above as, with the exception of Ingalhalikar et al. (2011), IQ in typically developing and ASC groups was unmatched or even unreported, despite the effects of this variable on white matter microstructure (Penke et al., 2012). Furthermore, some of these findings were obtained from non-verbal children, such that their specificity to language or to ASC in general remains unclear. To elucidate this specificity further, it would be important to study people with ASC who have intact language. To our knowledge, only two tractography studies to date have examined the arcuate in adolescents with high-functioning autism or Asperger syndrome. Whilst one study (Fletcher et al., 2010) revealed a lack of the typical structural lateralisation that corroborates the previous work by the Wan and Joseph groups, the other found no differences at all (McGrath et al., 2013; see Supplementary Materials for further details). These authors of the latter study note that they may have only analyzed a partial segment of the arcuate. This leaves open the question as to whether this or the high verbal ability of their participants resulted in the lack of differentiation between groups.

Given the small number of studies in this area and the limitations of previous work, the nature of putative structural changes to the arcuate fasciculus in autism is still largely unknown. Existing findings are divergent and sometimes contradictory, and this heterogeneity might have several sources. Previous studies have employed rather heterogeneous groups, differing in sex, age, and symptom severity. For example, the age range (and hence cognitive and general developmental stage) differs substantially from 5 (Joseph et al., 2014) to 14 years (Fletcher et al., 2010), making it difficult to compare data between studies. Moreover, childhood and adolescence are developmental periods involving substantial changes in structural and functional connectivity of the brain (Mukherjee et al., 2002; Nagy et al., 2004; Barnea-Goraly et al., 2005; Fair et al., 2009; Asato et al., 2010), which might be another reason for lack of arcuate difference in the McGrath study (McGrath et al., 2013). Even children of the *same* chronological age can show large differences in cognitive and social development (Fischer and Silvern, 1985), let alone those from such different age groups. Structural brain anatomy (including asymmetry) is modulated by biological sex in both typically developing individuals (Bao and Swaab, 2011) and those with ASC (Lai M.C. et al., 2012; Lai et al., 2013), which also has to be taken into account in any neuroanatomical study. Furthermore, many of the above studies (Ingalhalikar et al., 2011; Lai G. et al., 2012; Wan et al., 2012) tested children who were very low functioning with severely impaired language and very low verbal IQ. We therefore cannot ascertain whether these reported arcuate differences in low-functioning autism would be seen in children with autism who are verbal, or are only related to being non-verbal. In fact, Fletcher et al. (2010) failed to replicate these results in their sample of teenagers with ASC who had average full-scale and verbal IQ. Finally, the arcuate in an adult population of people with ASC have not been examined.

To fill these gaps, we aimed to investigate the structural integrity of the arcuate fasciculus in a homogenous group of high-functioning adults with ASC who did not show any intellectual disability or obvious language impairments. This group has been understudied in terms of structural differences in language-related fiber tracts. It is of interest to examine whether this population shows atypical features similar to those seen in individuals with clear language delays and deficits, which could then be attributed to core features of ASC rather than to the obvious language impairments manifest in the latter group. As individuals with Asperger syndrome show subtle linguistic abnormalities (Boucher, 2003; Eigsti et al., 2011), differences in the structural architecture of language can be predicted in this population. Based on previous findings, we were interested in measures of cortical asymmetry of the arcuate and in any differences in FA, mean diffusivity (MD) and volume between highly verbal adults with and without ASC. Expecting that microstructural differences of the arcuate might appear even in this high-functioning population, we also examined correlations between DWI measures and the AQ (Baron-Cohen et al., 2001), a measure of autistic traits, to see whether a dimensional relationship exists between autistic traits and arcuate structure.

## Materials and Methods

### Participants

Participants included 18 adults (mean age: 30.39 [standard deviation (SD): 9.99]; 10 males) with high-functioning autism or Asperger syndrome and 14 neurotypical adults (mean age: 27.64 [SD: 11.28]; 10 males). All participants were right-handed, native monolingual English speakers, medication-free, and none had a history of neurological disorder. Handedness was assessed using the Edinburgh Handedness Inventory (Oldfield, 1971), and IQ using the Cattell Culture Fair test. Demographics for all measures are shown in **Table 1** (see Results). All ASC subjects were verbally fluent without any obvious clinical manifestations of language abnormalities, although were previously shown to exhibit subtle differences in semantic processing under experimental conditions (Moseley et al., 2013, 2014, 2015). In the ASC group, participants demonstrated a high degree of functional adaptation, as indicated by their employment status. Ten participants were employed, five were studying at University and only three participants were unemployed. All participants had completed full time education.

**Table 1:** Participant demographics and statistical comparison of group averages for the mixed-gender sample.

	ASC group (*N* = 18)	Control group (*N* = 14)	Statistical testing (t)
Age	30.39 (9.99) [39]	27.64 (11.28) [44]	0.729, *p* = 0.472
Handedness	76.1 (26.2) [60]	90 (14.1) [40]	1.790, *p* = 0.085
IQ	112.72 (22.56) [66]	108.86 (12.67) [42]	0.573, *p* = 0.571
Autism-spectrum quotient (AQ)	34.9 (11.3) [35]	12.71 (5.6) [19]	6.722, *p* < 0.001

*Values represent group averages with standard deviations in brackets () and range in square brackets [].*

The ASC sample was recruited from the volunteer database at the Autism Research Centre at Cambridge University^1^. They had all been previously clinically diagnosed using DSM-IV criteria: 17 met criteria for Asperger Syndrome, and one for PDD-NOS. All completed the AQ. To account for the heterogeneity in our sample introduced by biological sex, a secondary analysis included only the 10 males in each group.

All participants gave written informed consent prior to participating in this study, indicating that they understood its purpose and were willing for their data to be included (in anonymous form) in scientific reports. They were remunerated for their time. Ethical approval was provided by NHS Research Ethics Committee of Cambridgeshire.

### Imaging and Statistical Analysis

Participants were scanned in a 3T Tim-Trio scanner, using a 12-channel head-coil. Whole brain DWI data was acquired [Repetition Time (TR) = 7800 ms, Echo Time (TE) = 90 ms, field of view: 19.2 cm, slice thickness: 2 mm, 63 slices, acquisition matrix size: 96 × 96, voxel size: 2 mm × 2 mm × 2 mm, GRAPPA acceleration factor of 2) using a twice refocused spin echo sequence to reduce eddy currents (Reese et al., 2003). Diffusion sensitizing gradients were applied along 64 gradient directions with a *b*-value of 1000 mm^2^/s. A high resolution T1-weighted MPRAGE scan was also acquired (TR = 2250 ms, TE = 2.99 ms, field of view: 256 mm × 240 mm, slice thickness: 1 mm, 192 slices, GRAPPA acceleration factor of 2).

For the purpose of estimating global white matter and ICV in participant MPRAGE (T1-weighted) files, preprocessing and segmentation of white and gray matter was performed using Freesurfer (Fischl, 2012), a well-documented analysis tool freely available online^2^. ICV was calculated by the automated ‘eTIV’ process within the mri_segstats function, which derives ICV through brain atlas normalization procedures that calculate head size (Buckner et al., 2004).

Motion parameters were extracted for each DWI volume for all participants using FSL’s motion and eddy current correction function eddy_correct^3^, and any participants who moved more than 2 mm in any direction were excluded. The diffusion weighted volumes were also visually inspected for typical motion artefacts (e.g., striping), but no further participants needed to be removed for this reason.

In order to check whether there was a difference in the amount of motion between the two groups (ASC vs. controls), a summary measure of motion was determined using the root mean square (RMS) volume of the six parameters describing the rigid body movement (three translations and three rotations). This summary measure was calculated both in absolute terms (i.e., using the firstly acquired volume as a reference), giving a global measure of head motion, and also relative to the preceding volume, giving a measure of the head motion between volumes. The average relative head displacement between volumes was 0.55 mm for the controls, and 0.58 mm for the ASC participants, while the average absolute displacement was 1.47 mm for the controls and 1.54 mm for ASC participants. There was no significant difference between groups (*p* = 0.47 for absolute displacement and *p* = 0.55 for relative displacement). The maximum relative and absolute displacement for each subject were also compared across groups and again no difference was found (*p* = 0.96 for absolute displacement and *p* = 0.41 for relative displacement).

Preprocessing and analysis of the DWI was conducted using MRtrix (J-D Tournier, Brain Research Institute, Melbourne, Australia^4^), and the full analysis was performed in subject-space. Initially, images were converted from DICOM to MRtrix (.mif) format. A brain-mask with the same dimensions as the diffusion dataset was generated for each participant for use in further analysis, and these were checked against the original DWI images in order to determine whether any manual edits of the mask were required. The diffusion tensor model was then fitted to the DWI data, and a map of FA was generated for each subject.

The arcuate was reconstructed using probabilistic fiber-tracking based on CSD (Jeurissen et al., 2011). The majority of previous diffusion MRI studies in ASC have used diffusion tensor imaging (DTI) to reconstruct white matter bundles of interest. However, a well known limitation of this approach is its inability to account for crossing fibers in the brain, and the CSD approach was therefore chosen in order to overcome this limitation. CSD is a very powerful tractography technique which is able to trace white matter bundles across regions of crossing fibers, while keeping the total acquisition time manageable for ASC participants (∼10 min). Other crossing-fiber reconstruction techniques, such as diffusion spectrum imaging (DSI), require significantly greater imaging times (>30 min), which makes them unsuitable for ASC studies due to the increased discomfort this would impose.

**FIGURE 1:**
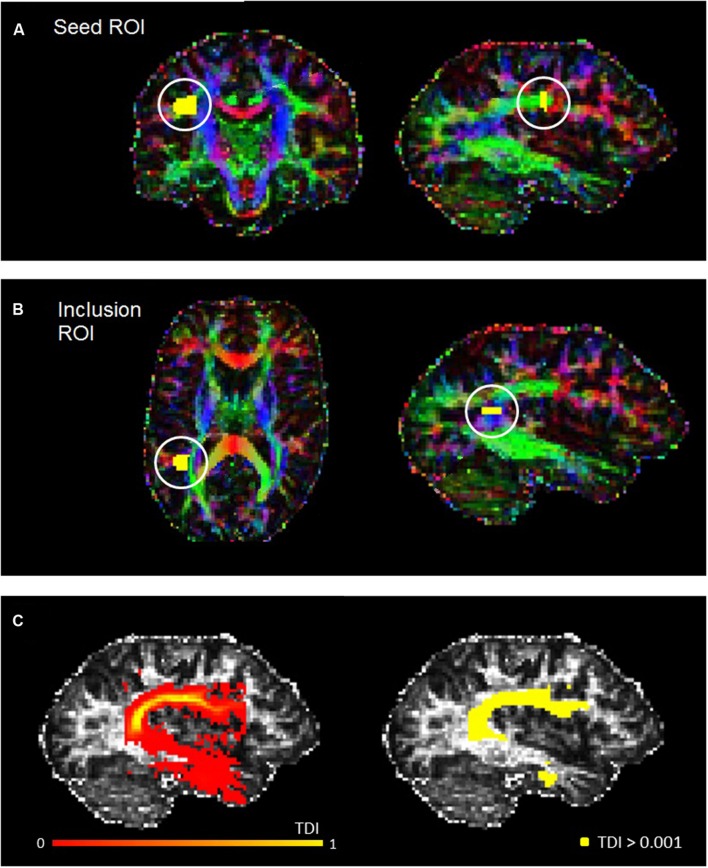
**Example seed (A) and inclusion (B) ROIs for a representative participant, defined in accordance with Liégeois et al. (2013). (C)** Shows the track-density image for the left AF of the same participant (left), and also the thresholded AF mask used for the statistical analysis (right).

The fiber orientation distribution function was estimated for each voxel, and a probabilistic fiber-tracking algorithm was used (Jeurissen et al., 2011). Probabilistic algorithms are regarded as less sensitive to noise or artefacts, and better able to account for uncertainty and to reconstruct areas of crossing fibers (Behrens et al., 2007; Klein et al., 2010). The masking and editing tool included in FSLview (Jenkinson et al., 2012) was used to draw seed and target regions of interest (ROIs) in the right and left hemisphere of each participant in native space (see **Figure 1**). The ROI drawing procedures implemented followed protocol for dissecting the arcuate fasciculus which were published by Liégeois et al. (2013), although for both ROIs we used two slices instead of three. Initially, a seed ROI was placed on two coronal slices at the so-called arcuate “bottleneck”: an anterior-posterior orientated fiber tract lateral to the corona radiata and medial to the cortex (see **Figure 1A**). All fibers must pass through this point to reach their destination, and so fibers were reconstructed between this seed and a second “inclusion” ROI, which was placed on two slices in the axial plane, corresponding to superior temporal gyrus (see **Figure 1B**). Only tracks which passed through this ROI were included. From these tracks, high-resolution TDI were generated and examined for spurious fibers. These were removed by manually creating exclusion ROIs and repeating the tracking protocol. The following exclusion ROIs were used when necessary: (1) an axial ROI to exclude descending cortico-spinal tracts; (2) an axial ROI above the AF to exclude ascending cortical tracts; (3) a coronal or sagittal ROI to exclude tracts belonging to the inferior longitudinal fasciculus; and, (4) a sagittal ROI to exclude tracts crossing between the hemispheres. All ROIs were drawn by RM, and subsequently checked and adjusted if necessary by MMC.

With spurious or curling fibers removed, we thresholded the TDI with an absolute intensity of 0.001 (see **Figure 1C**). This thresholded output was then used as a mask to run the ‘mrstats’ function, which calculated the volume of (number of voxels in) the binary arcuate fasciculus mask. The AF masks were also used to calculate average FA and MD along this tract for every participant. The former is a common indicator of microstructural integrity which reflects the degree of anisotropy in brain tissue: whilst low FA values indicate that diffusion of water molecules is restricted or unrestricted in all directions, higher values reflect diffusion that is highly directed along one axis. MD (known as apparent diffusion coefficient in some publications [Kumar et al., 2010]), which contributes to the calculation of FA, reflects the trace of the tensor and the magnitude of diffusion (Alexander et al., 2007).

The values for each participant were then entered into a statistical program (SPSS v.21) for analysis. One-level ANOVAs were initially performed to look for differences in participant demographics like age, IQ or handedness that might influence arcuate structure. Volume and FA of the arcuate were analyzed in two two-level ANOVAs with the factors Group (ASC vs. Controls) and Hemisphere (left vs. right hemisphere). Finally, we performed Pearson correlations to examine the relationship between FA, volume, and autistic traits (AQ scores).

## Results

### Pre-experiment Group Differences

Participant demographics and statistically significant group differences are reported in **Table 1**.

The two groups did not differ significantly in age, handedness or IQ, such that differences in arcuate structure could not be related to any of these variables. Though the ASC group were less strongly right-handed than controls, this was non-significant and a common feature of this population (Tsai, 1984).

As expected, a highly significant difference appeared in their AQ scores, which are generally strongly predictive of diagnostic status (Baron-Cohen et al., 2001; Woodbury-Smith et al., 2005; Hoekstra et al., 2008).

### Structural Imaging Analysis: Fractional Anisotropy (FA), Mean Diffusivity (MD) and Volume

**FIGURE 2:**
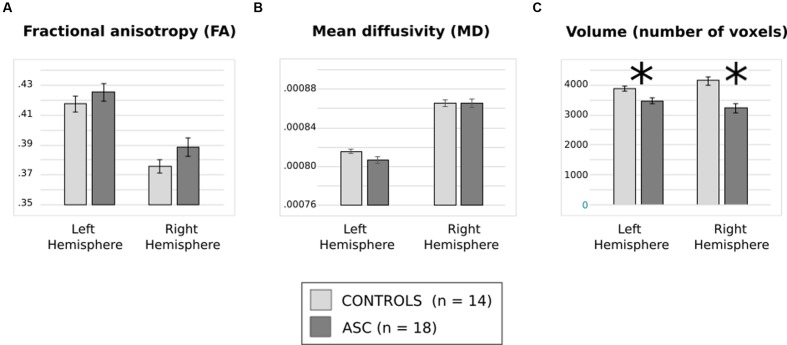
**(A)** Average fractional anisotropy (FA), **(B)** mean diffusivity (MD) and **(C)** volume of (number of voxels in) the arcuate fasciculus for each group. Error bars reflect standard error. Asterisks (^∗^) reflect significant group differences.

Analysis of FA revealed a significant main effect of Hemisphere [*F*_(1,30)_ = 130.112, *p* < 0.001], reflecting that both groups showed typical lateralisation patterns with greater FA in the left than the right hemisphere (see **Figure 2A**). Analysis of MD, too, showed a main effect of hemisphere reflecting rightward lateralization [*F*_(1,30)_ = 78.400, *p* < 0.001] but no effect of group and no interaction (**Figure 2B**).

There was a significant interaction of Group and Hemisphere for arcuate volume [*F*_(1,30)_ = 6.194, *p* = 0.019] and, in addition, a highly significant main effect of Group [*F*_(1,30)_ = 23.963, *p* < 0.001]. *Post hoc t*-tests revealed a significant relative reduction in the volume of the left [*t*_(30)_ = 2.985, *p* = 0.006] and the right [*t*_(30)_ = 4.557, *p* < 0.001] arcuate in the ASC group (see **Figure 2C**). A lack of any significant differences in global white matter volume (*p* = 0.453) showed that this was a specific rather than a global effect. Within-group tests showed that although ASC participants showed no significant volumetric differences between the left and the right hemisphere, control participants actually showed greater volume in the right arcuate [*t*_(13)_ = 2.654, *p* = 0.020], though both groups were left-lateralized for FA.

### Correlation of Arcuate Structure and Clinical Measures

**FIGURE 3:**
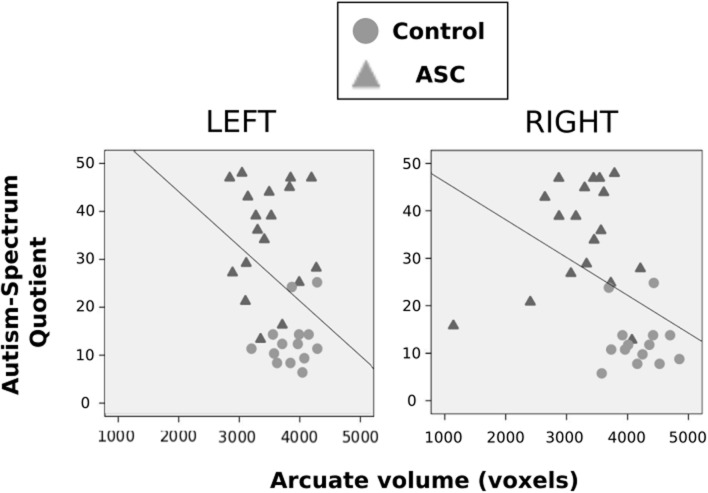
**Correlations between autistic traits, as measured by the AQ, and volume of the arcuate fasciculus.** These are displayed for the left and right hemispheres, respectively, with control participants represented by gray circles, ASC participants by gray triangles.

Using Pearson correlations in all participants pooled, we found that AQ scores negatively correlated with volume of the right (*r* = -0.413, *p* = 0.019) arcuate, with a similar marginal trend in the left hemisphere as well (*r* = -0.342, *p* = 0.056). In both cases, a greater number of autistic traits was associated with reduced volume in the arcuate fasciculus (see **Figure 3**). This correlation fell beneath significance when examined in each group independently. Neither FA or MD in either hemisphere correlated with autistic traits.

### Male-only Analysis

Sex is a major confound in mixed-gender samples, given that males typically have larger heads than females and thus have greater general ICV. This was true in the current sample of males and females [*t*_(30)_ = 3.134, *p* = 0.004], and by virtue of the fact that we recruited more females with ASC than previous studies in this field, the ASC group had significantly lower ICV [*t*_(30)_ = -2.147, *p* = 0.04] than controls. Multiple regression analyses revealed that whilst ICV contributed to predict left arcuate volume (*B* = 180.864, *t* = 2.495, *p* = 0019), it did not significantly predict right arcuate volume (*B* = 34.926, *p* = 0.839, *p* = 0.408). Indeed, adding ICV as a covariate in our statistical tests showed that the Hemisphere by Group interaction remained significant [*F*_(1,29)_ = 6.060, *p* = 0.020], as did the main effect of Group [*F*_(1,29)_ = 16.411, *p* < 0.001]. As an additional step to confirm this, we normalized arcuate volume for ICV (i.e., dividing arcuate volume in each subject by ICV): the Hemisphere by Group interaction [*F*_(1,30)_ = 5.774, *p* = 0.023] and Group effect [*F*_(1,30)_ = 5.350, *p* = 0.028] remained significant, as did the group difference in the right hemisphere [*t*_(30)_ = 2.732, *p* = 0.01], but the group difference in the left hemisphere became robustly non-significant (*p* = 0.512).

We repeated our analysis with a reduced, sex-matched sample, a recommended strategy on the basis of neuroanatomical differences between the sexes (Lai et al., 2013). This time, the groups (10 males in each) were matched not only in global white matter volume [*t*_(18)_ = 0.909, *p* = 0.375] but also in ICV [*t*_(18)_ = 0.536, *p* = 0.536]. They also remained matched in all their demographic data, as can be seen below (**Table 2**).

**Table 2:** Participant demographics and statistical comparison of group averages for the reduced, all-male sample.

	ASC group (*N* = 10)	Control group (*N* = 10)	Statistical testing (t)
Age	32.8 (11.11) [34]	29.1 (12.9) [44]	0.515, *p* = 0.613
Handedness	76 (30.6) [60]	90 (12.5) [60]	1.339, *p* = 0.197
IQ	112.3 (26.7) [60]	107.5 (12.5) [42]	0.684, *p* = 0.502
Autism-spectrum quotient (AQ)	32.5 (9.1) [29]	13.8 (6) [16]	5.438, *p* < 0.001

*Values represent group averages with standard deviations in brackets () and range in square brackets [].*

**FIGURE 4:**
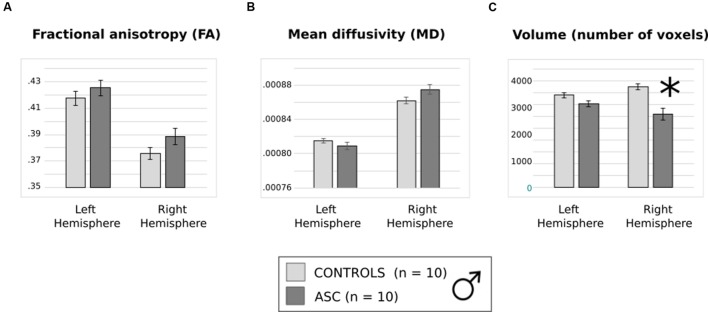
**(A)** Average fractional anisotropy (FA), **(B)** mean diffusivity (MD) and **(C)** volume of (number of voxels in) the arcuate fasciculus in the smaller, male only group. Error bars reflect standard error. Asterisks (^∗^) reflect significant group differences.

Previous trends in FA and volume remained consistent in this smaller subset. Though FA and MD did not differ between groups (**Figures 4A,B**), a main effect of hemisphere reflected that both had higher FA in the left than the right arcuate [*F*_(1,18)_ = 77.978, *p* < 0.001] and higher MD in the right than the left arcuate [*F*_(1,18)_ = 46.404, *p* < 0.001]. The two-factor ANOVA of volume revealed a significant Hemisphere by Group interaction [*F*_(1,18)_ = 7.820, *p* = 0.012] and a main effect of Group [*F*_(1,18)_ = 16.287, *p* = 0.001]. Just as before, the ASC group showed significant reduction in the volume of the right arcuate as compared with controls [*t*_(18)_ = 16.669, *p* < 0.001], though their reduction in the volume of the left arcuate became marginally non-significant [*t*_(18)_ = 2.041, *p* = 0.056] (**Figure 4C**). Within groups, the ASC participants showed no significant volumetric differences between the left and the right arcuate, but the typically developing participants showed greater volume in the right than the left arcuate [*t*_(9)_ = 2.736, *p* = 0.023]. Although the male groups were matched in ICV, we added this as a covariate in our tests to ensure that results did not change substantially. Indeed, there was little effect on the Group by Hemisphere interaction [*F*_(1,17)_ = 7.114, *p* = 0.016] or the Group effect [*F*_(1,17)_ = 15.576, *p* = 0.001].

Similarly to the main analysis, correlation tests were performed on these male participants pooled. Once again, with all participants pooled, higher AQ scores correlated with lowest volume in the right arcuate fasciculus (*r* = -0.478, *p* = 0.033). Correlations with AQ were not significant for either of the male groups alone.

## Discussion

Probabilistic tractography revealed a significant volumetric reduction of the arcuate fasciculus, an effect strongest in the right hemisphere, in high-functioning individuals with ASC as compared with typical controls. Although this result could in part be attributed to group differences in ICV, multiple regression of ICV did not appear to contribute significantly to right arcuate volume and, crucially, analysis of male participants only confirmed these volumetric differences in groups matched for ICV.

Furthermore, significant correlations revealed a negative relationship between right arcuate volume and the presence of autistic traits as revealed by the AQ. This shows that decreased volume of the right arcuate is associated with a higher number of autistic traits related to social interaction, lack of imagination, empathy, restricted interests and obsessions, and repetitive behavior. However, when correlations between arcuate volume and autistic traits were performed separately for the mixed and male ASC groups and the control group, the correlation was not significant for any group. This may be due to the rather small size of each group, making the statistical power insufficient for separate analyses. It could, however, reflect that the correlation in all subjects pooled was driven by the group difference seen between individuals with and without ASC. Replication of results in a larger sample would certainly be required in order to confirm a relationship between dimensional autistic traits in the distribution of the normal population and the volume of the arcuate fasciculus.

### The Arcuate in Autism: Placing Our Findings in Context

Our findings contribute to a small literature on the subject of structural changes in the arcuate fasciculus in autism. Our present findings converge with all previous studies in showing that the structure of this major language pathway is altered in high- and low-functioning ASC (although see McGrath et al., 2013 for a divergent view). However, we should also highlight some divergence, if not incompatibility, between the present findings and those of earlier work.

Investigations of FA report inconsistent results across the literature: previous studies have reported generally lower FA in ASC as compared to typically developing controls (Kumar et al., 2010; Lai G. et al., 2012), just relatively reduced laterality of FA in ASC (Fletcher et al., 2010), or even no differences in FA between groups at all (Joseph et al., 2014). Our findings correspond with the latter finding: both groups showed the typical left-hemispheric lateralisation of FA and did not differ significantly from each other in this measure. Of these previous reports of altered FA, however, only one reports any slight difference in a highly verbal group (Fletcher et al., 2010). We did not see a difference in the lateralisation of FA, and so further research is needed to reconcile these two reports, which could potentially relate to the different ages of ours and the Fletcher group’s samples (see below).

Autistic and control groups did not differ in MD but instead exhibited a rightward laterality which some groups have suggested may be common in typically developing individuals (Fletcher et al., 2010). Two previous studies also failed to find differences between children with ASC and typically developing peers in MD (Ingalhalikar et al., 2011; Joseph et al., 2014). Another reported an increase in right-hemispheric MD in children with ASC, but in this variable the group did not differ from children with non-specific developmental impairments (Kumar et al., 2010). In high-functioning participants, Fletcher et al. (2010) found reduced hemispheric *asymmetry* in MD, but did not compare MD directly between groups. Differences in MD are certainly not a strong feature of the landscape in studies investigating the arcuate in autism.

Like FA, findings related to volume have been similarly inconsistent. It should be said that, just as fMRI is an indirect measure of neuronal activity, this measure implies reduced connectivity but cannot directly indicate that the existing tissue is compromised. Group differences are absent in some studies (Fletcher et al., 2010). Other studies with low-functioning children report reduced left-lateralization in autism (Wan et al., 2012; Joseph et al., 2014). Our ASC sample showed slightly greater volume in the left than the right arcuate, but like these studies, we did not see the significant left-lateralization of the arcuate in controls which has been reported in previous research with typically developing participants.

This is, at first glance, an unusual finding. Individual variability in structural (Catani and Mesulam, 2008) and functional (Lidzba et al., 2011) lateralisation does occur, but it may be important at this point to consider differences in the delineation of the arcuate which may contribute to differences in lateralisation of arcuate volume and structure. Although it is widely accepted that the ‘arcuate’ is left-lateralized, there may be conceptual confusion in the field regarding exactly which white matter tracts are delineated as ‘arcuate fasciculus.’ Some researchers (Catani et al., 2005, 2007; Catani and Thiebaut de Schotten, 2008) have subdivided the arcuate into three segments: a direct segment connecting Wernicke’s and Broca’s territories (posterior inferior frontal cortex and posterior temporal cortex, respectively), an anterior indirect segment connecting Broca’s territory to inferior parietal cortex, and a posterior indirect segment connecting Wernicke’s territory to inferior parietal cortex. These authors do not differentiate the arcuate from the SLF, though the protocol which we follow defines it as part of a “dorsal pathway[…] the long segment of the SLF that connects Broca’s and Wernicke’s areas” (Liégeois et al., 2013). The established differentiation between SLF and the arcuate is highlighted by Makris et al. (2005), who also recommend splitting the SLF into four tracts (SLF I, II, III, and the arcuate). These authors suggest that what Catani et al. (2005, 2007) conceptualize as the anterior indirect (frontoparietal) arm of the *arcuate* is in fact a separate branch of the inferior SLF (segment III). The arcuate in their narrower sense, that is the “direct” frontotemporal segment of this pathway, runs closely alongside the “indirect” frontoparietal section (“SLF III”), such that differentiation between the two (and equally between the arcuate and parieto-temporal short segment), if desired, is challenging. If we adopt the Catani definition of the arcuate (including ‘direct’ and ‘indirect’ segments), closer examination reveals that as a whole, the volume of the arcuate fasciculus is *not* strongly left-lateralized. Although the *direct long frontotemporal SLF* segment has indeed been reported to be left-lateralized in FA and volume, the arcuate as a whole is slightly right-lateralised in volume and left-lateralized in FA (Thiebaut de Schotten et al., 2011), a pattern consistent with what we observed in our typically developed controls.

With no a priori hypothesis predicting differences in particular *segments* of the arcuate, we employed the approach of greatest familiarity to our group [that employed by Liégeois et al. (2013)], and so our procedures for fiber definition, which focussed on temporal and parietal ROIs (see Materials and Methods), may have led to inclusion of both the long fronto-temporal segment as well as part of the short parieto-temporal segment of the arcuate. Variation in tracking protocols for arcuate delineation may contribute to heterogeneity in results between ASC studies. Whilst some studies employed the Catani protocols (Wan et al., 2012) or placed seed ROIs in the same approximate locations (Fletcher et al., 2010; McGrath et al., 2013) as in the current study, others, for example, approximated the arcuate from dorsal projections from primary auditory cortex (Lai G. et al., 2012).

There are several other reasons for inconsistencies across studies, all of which make comparison difficult. Some of these include (1) discrepant language ability of participants, particularly given that presence or absence of childhood language delay (irrespective of current language) modulates brain structure (Lai et al., 2014), and (2) the age of participants (since many previous arcuate studies investigated children or adolescents vs. the adult group here). The most comparable study is that of McGrath et al. (2013), who studied highly verbal adolescent boys and used a similar placing of ROIs to delineate the arcuate. These authors did not find differences in the arcuate, but still examined significantly younger individuals (mean age: 17.37 in ASC) than the present study did (mean age: 30.39 in ASC). Joseph et al. (2014) found no relationship between age and their structural arcuate measures (volume, FA, mean, radial, or axial diffusivity), but with the extremely small age range of the sample, data on the relationship between age and arcuate structure in this study is not sufficient to allow clear-cut conclusions to be drawn on this issue. In a large sample including a total of 241 children, Su et al. (2008) report differences in myelination speed of language-related brain structures across the lifespan with slowest maturation of AF fiber tracts. These data indicate that any differences between previous studies in ASC children and our study can be strongly influenced by the myelination of the AF. Interestingly, recent large-scale investigations in infants with ASC suggest that the developmental trajectory of the arcuate may be substantially different from as early as 12 months of age (Solso et al., 2014). Researchers have called for a developmental perspective in studies of *functional* connectivity in autism (Uddin et al., 2013). Likewise, longitudinal research with large samples may be needed to validate the relationship between neuroanatomical correlates of the arcuate and age in children and adults with ASC, and might benefit from DWI sequences with higher angular resolution.

Apart from age and methodological issues, sex is a factor that seems to play a certain role in brain structure and function. Unfortunately, in our sample, we did not have enough female participants in each group to investigate FA, MD and volume of the arcuate fasciculus in well-matched female groups. As women with autism appear to exhibit markedly different neuroanatomical profiles compared to males (Lai G. et al., 2012; Lai et al., 2013), further research is needed to ascertain whether they also show volumetric arcuate reductions in comparison with typical females. Moreover, factors such as functional laterality and language ability should be assessed in larger group samples as these factors systematically differ between males and females (Caplan and Dapretto, 2001; Good et al., 2001; Eckert and McConnell-Ginet, 2003).

Our findings may constitute a profile for an under-studied group, verbal high-functioning male *adults* with ASC, and should be considered in this context. The crucial finding, in our view, is that despite their high-functioning diagnostic status, these individuals still exhibit a quantitative difference in arcuate volume compared to typical controls. As they are matched to typical controls in IQ, autistic traits are not here confounded by lower mental ability as they have been in previous studies (Kumar et al., 2010; Ingalhalikar et al., 2011; Lai G. et al., 2012; Wan et al., 2012; Joseph et al., 2014), and so alterations in arcuate structure can be more confidently ascribed to the ASC phenotype. Nevertheless, further research on the arcuate is needed to validate these volumetric differences and the lack of differentiation in FA in this small, highly verbal segment of the autism spectrum.

### Language Functions of the Right Hemisphere

Perhaps surprisingly, the reduction in arcuate volume that we observed in ASC was more striking in the right hemisphere than the left: this was reflected in the interaction of Hemisphere and Group that we observed in both the mixed sex and males only analyses. A strongly significant group difference in left arcuate volume seemed to be driven by differences in ICV and became marginally non-significant (*p* = 0.056) in the male group alone. In contrast, the significance of the difference on the right even survived after exclusion of females. Whilst the marginal effect in the left hemisphere still suggests a trend toward general reduction of this language pathway, it leads us to speculate on the particular role that the right hemisphere plays in language processing and the language differences in autism, especially given the association between AQ and right arcuate volume that we observed.

Despite the well-reported left-lateralization of language (Gazzaniga, 2000), optimal linguistic function requires the cooperation of both cerebral hemispheres (Mohr et al., 1994). Right-hemispheric involvement in language processing includes semantics (Pulvermüller and Mohr, 1996; Pulvermüller, 1999), and morphology (Marslen-Wilson and Tyler, 2007), but most notable is its role in social and pragmatic aspects of language (Coslett and Monsul, 1994; Zaidel, 1998; Mitchell and Crow, 2005; Lindell, 2006). The right hemisphere is crucial for production and comprehension of emotional prosody (George et al., 1996; Ross et al., 1997; Baum and Pell, 1999; Buchanan et al., 2000; Wildgruber et al., 2009), non-literal language such as metaphors (Brownell et al., 1990; Tompkins, 1990; Bottini et al., 1994), jokes (Shammi and Stuss, 1999), and indirect requests (Foldi, 1987). These abilities intersect closely with theory of mind, the ability to infer a speaker’s or listener’s intentions and current knowledge. The right hemisphere is also crucially involved in resolving lexical ambiguity (Burgess and Simpson, 1988), drawing figurative inferences from language (Nichelli et al., 1995), processing its broader context (Caplan and Dapretto, 2001), and performing and comprehending socio-communicative ‘speech acts’ (Egorova et al., 2014) – all functions which make the right hemisphere absolutely essential for comprehending and smoothly contributing to discourse (Bryan, 1988; Schneiderman et al., 1992; Myers and Brookshire, 1996; Robertson et al., 2000; Zaidel et al., 2002). These pragmatic abilities, again, involve central coherence and sound understanding of the listener’s knowledge and mental state.

Consistent with our findings, the right arcuate fasciculus has been implicated previously in autism. As noted above, Kumar et al. (2010) found increased fiber length in the right arcuate fasciculus to set children with autism apart from typically developing and developmentally impaired children without autism. Increased fiber length does not appear to correspond with our finding of *reduced* right arcuate volume, but here we might consider the possible effects of age. There is an emerging view of ASC that hyperconnectivity in early life is reversed in adolescence, with hypoconnectivity more commonly reported in adulthood (Uddin et al., 2013; Nomi and Uddin, 2015). We speculate that this could be reflected here at a local level.

This study relied on previous diagnostic assessments that had established intact language development (i.e., no delay) in our participants. We can, however, still consider the *type* of language features that are typical of high-functioning individuals such as our sample. All the ASC participants were currently or had previously worked or studied. All but one (PDD-NOS) were clinically diagnosed with Asperger syndrome, which is differentiated from high-functioning autism on the basis of intact (no delay) development of language. This diagnostic distinction, however, is problematic (Frith, 2004; Bennett et al., 2008) and thus is no longer included in the DSM-V (American Psychiatric Association, 2013). Linguistic anomalies in high-functioning autism and Asperger syndrome are subtle but have been observed (Boucher, 2003; Eigsti et al., 2011). In addition, some language functions seen as right-hemispheric, such as comprehension and production of emotional prosody (Fine et al., 1991; Korpilahti et al., 2007), are atypical in these populations. Pragmatic impairments, such as in understanding jokes and discourse, are the most universal linguistic impairment in ASC (Landa, 2000; Colle et al., 2008; Groen et al., 2008; Eigsti et al., 2011). Semantic impairments are also present across the spectrum (Boucher, 2003; Groen et al., 2008; Eigsti et al., 2011), ranging from moderate to mild even in high-functioning autism and Asperger syndrome (Moseley et al., 2013, 2014, 2015), and the right arcuate has been particularly implicated in the semantic domain as well as that of prosody (Catani et al., 2007; Catani and Mesulam, 2008), although it certainly also carries phonological/lexical function (Berthier et al., 2012). We hypothesize that the rightward lateralisation of volumetric differences in our study reflect the typically right-hemispheric language impairments that high-functioning individuals may exhibit.

Given the good language capacities of our participants, it is therefore unsurprising that we did not replicate the findings from previous studies of low-functioning children (Lai G. et al., 2012; Wan et al., 2012). Quite aside from the fact that both studies tested young children who obviously are not comparable to adults, participants in the Wan study in particular were non-verbal. They reported an atypical pattern of asymmetry in their children, who showed greater volume of the right than the left arcuate. The analysis was based on calculation of ‘laterality index’ (numeric difference between left and right arcuate volume, divided by their sum), i.e., a relative measure, rather than direct volume comparison. Visual inspection of the figures suggests that there might be a difference in only the volume of the left arcuate fasciculus, which is larger in typically developing children than children with autism. The left-hemispheric difference may therefore reflect the linguistic disability of that sample. As the study did not include a comparison of verbal children with autism and typically developing controls, or a comparison with another non-verbal group, it is impossible to ascertain whether this difference is autism-specific or reflects the difference in language ability between *any* verbal and non-verbal children. Lai et al. (2014) recently demonstrated that even in high-functioning autism samples, the presence or absence of language delay is associated with substantial changes in gray and, to a lesser extent, white matter. An important direction for future research in this area would be to categorize autistic individuals on the basis of language delay or impairment, rather than diagnostic label, to compare the effect of high and low verbal ability on the structural properties of the arcuate fasciculus.

### The Specificity of Arcuate Abnormality

While we focus here on the structural hypoconnectivity of the arcuate, we stress that caution should be exercised regarding the specificity of ASC hypoconnectivity to this tract. No difference was seen in global white matter volume between our groups, which suggests specificity of the arcuate finding. This is not, however, a sufficiently rigorous test of structural integrity in other brain tracts, which might be differentially affected in autism. It is additionally important to reiterate again that volume is an indirect indicator of hypoconnectivity; that is, although the arcuate is smaller in ASC, we cannot conclude here that connectivity (at a functional or structural level) is compromised, although this interpretation would be consistent with a body of work reporting hypoconnectivity in ASC (see below).

It is difficult to comment on the specificity of the arcuate difference in the earlier research considered above. Wan et al. (2012) only defined the arcuate fasciculus in their participants and made no statements about specificity. Other researchers (Fletcher et al., 2010; Joseph et al., 2014) suggest specificity of arcuate hypoconnectivity: like us, both studies included a measure of global white matter volume which did not differ between groups. This, however, may not constitute a sufficiently adequate analysis of other tracts. Lai G. et al. (2012) identified dorsal and ventral tracts which originated from primary auditory cortex (A1, Heschl’s gyrus): the dorsal pathway was identified as the arcuate fasciculus, and the ventral pathway connected frontotemporal cortices via the extreme capsule, inferior fronto-occipital fasciculus and uncinated fasciculus. They found decreased FA in the left arcuate, but no microstructural differences in the ventral tract: somewhat limited evidence of specificity.

Ingalhalikar et al. (2011) attempted to classify subjects based on DWI anisotropy and diffusivity values. The brain regions contributing to diagnostic prediction included the left SLF (which includes the arcuate) but also the right internal and external capsule, the fornix, and white matter of the occipital gyri and inferior temporal cortex. McGrath et al. (2013), who failed to find arcuate differences in ASC, found differences in the inferior fronto-occipital fasciculus, though they did not examine any other tracts. Kumar et al. (2010) reported abnormalities of the corpus callosum, uncinate fasciculus *and* the arcuate which were specific to children with autism.

Specificity of hypoconnectivity to the arcuate fasciculus may be unlikely given the large body of work documenting atypical connectivity in autism in general (Kana et al., 2011; Müller et al., 2011; Uddin et al., 2011; Vissers et al., 2012; Di Martino et al., 2014). ASC have been described as “developmental disconnection syndromes” (Geschwind and Levitt, 2007), but in reality present a more complex and, as mentioned, sometimes heterogeneous neuroanatomical profile. Analyses of structural connectivity have reported differences in the corpus callosum (Frazier and Hardan, 2009; Booth et al., 2011) and white matter reductions in frontal, temporal and limbic cortices (Barnea-Goraly et al., 2004; Sundaram et al., 2008; Ecker et al., 2010). Contrary to these data, some studies report white matter excess, particularly in frontal cortex and locally, in the microcolumns of the brain (Herbert et al., 2004; Courchesne and Pierce, 2005; Mostofsky et al., 2007; Casanova and Trippe, 2009; Ecker et al., 2010; Weinstein et al., 2011). However, with a strict interpretation of ‘long-range’ connectivity as tracts connecting brain regions further than 1 cm apart, our findings corroborate the common view that atypical connectivity in ASC leans toward hypo-, rather than hyper-, connectivity in adulthood (Vissers et al., 2012).

Further research must investigate directly the contribution of arcuate abnormalities to autistic symptomatology, particularly those symptoms related to language.

## Conclusion

This study demonstrates structural, volumetric abnormalities in the arcuate fasciculus in high-functioning (verbal) individuals with ASC who have no apparent language difficulties and, in the case of those individuals with Asperger syndrome (94% of this sample), no delay in language development. Volumetric reductions of the arcuate tended to be present bilaterally but most strongly expressed and significant in the non-dominant right hemisphere, where they seemed to predict the severity of autistic symptoms. We suggest that the right-lateralised structural changes in the arcuate may constitute the neuroanatomical substrate of more subtle pragmatic and semantic language impairments seen in high-functioning individuals.

## Author Contributions

FP, BM, and RM were involved in initial experiment design. Recruitment of participants, collection of data, tractography and drawing of ROIs, statistical analysis and manuscript production were carried out by RM. MC guided RM in DWI analysis, checked, adjusted and validated ROIs drawn by RM, and contributed to the manuscript. BM provided theoretical input, assisted with participant recruitment, and contributed to the manuscript. SB-C assisted with participant recruitment, provided analysis advice and contributed to the manuscript. Both YS and FP supervised and advised RM during analysis and contributed to the manuscript, and BM and FP led the original conception of the study. All authors read and approved the final manuscript.

## Conflict of Interest Statement

The authors declare that the research was conducted in the absence of any commercial or financial relationships that could be construed as a potential conflict of interest. The reviewer H-YL and handling Editor declared their shared affiliation, and the handling Editor states that the process nevertheless met the standards of a fair and objective review.

## Footnotes

1 www.autismresearchcentre.com

2 http://surfer.nmr.mgh.harvard.edu/

3 www.fmrib.ox.ac.uk/fsl

4 http://www.brain.org.au/software/

## Abbreviations

ASC: Autism spectrum conditions
AQ: Autism-Spectrum Quotient
CSD: Constrained spherical deconvolution
DSM-IV-TR: Diagnostic and Statistical Manual of Mental Disorders IV, Text-Revised
DWI: diffusion-weighted imaging
FA: fractional anisotropy
ICV: intracranial volume
IQ: Intelligence Quotient
PDD-NOS: Pervasive Developmental Disorder Not Otherwise Specified
ROI: Region of interest
TDI: Track-density images
