# Supplementary material for: Reduced Volume of the Arcuate Fasciculus in Adults with High-Functioning Autism Spectrum Conditions
Source: Front Hum Neurosci. 2016 May 12;10:214. doi: 10.3389/fnhum.2016.00214 (PMC4867673; doi:10.3389/fnhum.2016.00214)
Supplement: Supplementary file 1 [file Table_1.DOCX]

Volumetric hypoconnectivity of the arcuate fasciculus in adults with high-functioning autism spectrum conditions

Supplementary materials

**Table 1: summary of previous structural investigations of the arcuate fasciculus in ASC.** Average sample demographics are given in columns two and three (number of males and standard deviations in brackets), sample matching information in column five, measures of arcuate microstructure in column six, and major findings summarised in column seven.

| **Authors (year)** | **ASC participant demographics** | **Typically-developing participant demographics** | **Other non-ASC control groups** | **Group matching and verbal ability** | **Measures of arcuate microstructure** | **Major arcuate findings in ASC** |
| --- | --- | --- | --- | --- | --- | --- |
| ***Studies of ASC populations with low language ability (or unmatched with controls in IQ)*** | | | | | | |
| Kumar et al. (2010), *Cerebral Cortex* | N = 32 (29 males); age = 5 (SD not provided, but range = 2.5-8.9 years). | N = 16 (12 males); age = 5.5 (SD not available, but range = 2.5-8.6 years). | Children with non-specific developmental impairments; N = 12 (10 males); mean age = 4.6 (SD not provided, but range = 3-9 years). | Matched in age and gender. No information on IQ or language ability of the ASC group; typical controls had “intellectual functioning within normal limits”. | FA, apparent diffusion coefficient (also known as ‘mean diffusivity’, the term we employ here), average fibre length, fibre volume, fibre density, and lengths of individual fibre tracts (fibre length profile) compared between groups. | - Decreased FA in left arcuate and increased diffusion coefficient (mean diffusivity) in the right arcuate; ASC and developmentally-impaired children did not differ in these measures. - Reversed asymmetry in average fibre length of the arcuate (i.e. greater fibre length in the right hemisphere, as compared with in the left hemisphere for typically-developing and developmentally-impaired children). - Fibre length profile suggested more long fibres in the right arcuate in ASC. |
| Wan et al. (2010), *Ann. N. Y. Acad. Sci.* | N = 5 (3 males); age = 6.7 (SD = 1.2). | N = 5 (3 males); age = 7 (SD = .09). | None. | Matched in age.  IQ not provided, but participants with ASC were non-verbal (defined as “the complete absence of intelligible words”). | Volume, “laterality quotient” (left volume - right volume)/(left volume + right volume). Non-parametric measures. | - Greater volume in the right than left arcuate (statistical significance not reported). |
| Ingalhalikar et al. (2011), *NeuroImage* | N = 45 (42 males); age = 10.5 (SD = 2.5). | N = 30 (14 males); age = 10.3 (SD = 2.5). | None | Matched in age. Nonverbal IQ is not provided, though authors claim that participants are matched. Language abilities of the ASC group are heterogeneous, with 13 participants described as language-impaired.  Authors provide scores on the Social Communicative Questionnaire (relevant to communication skills) and the CELF-4 (Clinical Evaluation of Language Fundamentals – edition 4), a measure of higher-order expressive and receptive language functions (e.g. grammar, syntax, semantics). Typically-developing participants significantly higher in these measures. | FA, mean diffusivity of the superior longitudinal fasciculus, which includes the arcuate. | - Reduced FA in the left superior longitudinal fasciculus. |
| Lai et al. (2012), *Brain* | N = 16 (14 males); age = 11.02 (SD = 3.7). | N = 18 (14 males); age = 11.17 (SD = 4.4). | None. | Matched in age. IQ not provided, but authors state that IQ was unequal between groups as children with ASC were low-functioning. Language ability of ASC sample was extremely low as measured in diagnostic tests, with little spontaneous speech. | Identification of ‘dorsal’ (arcuate) and ‘ventral’ tracts connecting a seed in primary auditory cortex to inferior frontal gyrus. FA, mean diffusivity, tensor norms, and analysis of termination location of ‘dorsal’ and ‘ventral’ tracts. | - Lower FA in the left ‘dorsal’ tract. |
| Joseph et al. (2014), *Brain Imaging and Behaviour* | N = 20 (18 males); age = 5.11 (SD = 1.3); verbal IQ = 96 (SD = 23); nonverbal IQ = 99 (SD = 21).  Demographics supplied for 20 participants, but only 16 used in DTI analysis. | N = 20 (15 males); age = 9.4 (SD = 1.3); verbal IQ = 117 (SD = 15); nonverbal IQ = 113 (SD = 11).  Demographics supplied for 20 participants, but only 16 used in DTI analysis. | None. | No matching data provided. ASC children had “at least phrase speech”. Two measures of language ability scores devised by 1) averaging oral language measures, 2) averaging this value and verbal IQ. No matching data for language ability. | FA, mean diffusivity, axial diffusivity, radial diffusivity, volume. | - Significantly lower volume in the left arcuate. - Larger left than right volume in the control group, but no between-hemisphere differences in ASC. Both groups showed left-lateralisation of FA; no differences in mean or axial diffusivity. - Radial diffusivity significantly higher in the left than the right hemisphere in controls; no differences in radial diffusivity between hemispheres in the ASC group. |
| ***Studies in ASC populations with high language ability (or matched with control groups)*** | | | | | | |
| Fletcher et al. 2010, *NeuroImage* | N = 10 (10 males); age = 14.25 (SD = 1.9); performance IQ = 108.8 (SD = 13.9); verbal IQ = 103.7 (SD = 18.6); handedness = 80.6 (SD = 19.1); head circumference (cm) = 55 (SD = 1.8). | N = 10 (10 males); age = 13.36 (SD = 1.3); performance IQ = 112.5 (SD = 14.8); verbal IQ = 102.7 (SD = 9.5); handedness = 81.30 (SD = 14.3); head circumference (cm) = 55.7 (SD = 1.6). | None. | Matched in age, performance IQ, verbal IQ, handedness, and head circumference.  ASC group significantly lower language abilities on the CELF-3. | FA, mean diffusivity, axial diffusivity, radial diffusivity, and volume. | - Abnormal lateralisation of mean and radial diffusivity (typically lower in the left, whereas ASC lack this effect). No group differences in volume, FA or axial diffusivity. |
| McGrath et al. (2013), *Autism Research* | N = 25 (25 males); age = 17.3 (SD = 2.9); IQ = 106.8 (SD = 14.5). | N = 25 (25 male); age = 17.4 (SD = 2.7); IQ = 110.7 (SD = 16). | None. | Matched in age and IQ. | FA, planar diffusion coefficient, linear diffusion coefficient. | - No group differences in FA, planar or linear diffusion coefficients, or lateralisation of any of these measures. |
